# The role of Serpina3n in the reversal effect of ATRA on dexamethasone-inhibited osteogenic differentiation in mesenchymal stem cells

**DOI:** 10.1186/s13287-021-02347-0

**Published:** 2021-05-17

**Authors:** Hai-Tao Jiang, Rui Deng, Yan Deng, Mao Nie, Yi-Xuan Deng, Hong-Hong Luo, Yuan-Yuan Yang, Na Ni, Cheng-Cheng Ran, Zhong-Liang Deng

**Affiliations:** 1 Department of Orthopaedics, The Second Affiliated Hospital of Chongqing Medical University, 76 Linjiang Road, Yuzhong District, Chongqing, 400010 China; 2 Chongqing Key Laboratory of Biochemistry and Molecular Pharmacology, Chongqing Medical University, No. 1 Yixueyuan Road, Yuzhong District, Chongqing, 400010 China; 3 Department of Pharmacology, School of Pharmacy, Chongqing Medical University, No. 1 Yixueyuan Road, Yuzhong District, Chongqing, 400010 China; 4 Ministry of Education Key Laboratory of Diagnostic Medicine, School of Laboratory Medicine, Chongqing Medical University, No. 1 Yixueyuan Road, Yuzhong District, Chongqing, 400010 China

**Keywords:** Glucocorticoid-induced osteoporosis (GIOP), Dexamethasone, ATRA, Serpina3n, BMP9, Osteogenic differentiation

## Abstract

**Background:**

Glucocorticoid-induced osteoporosis (GIOP) is the most common secondary osteoporosis. Patients with GIOP are susceptible to fractures and the subsequent delayed bone union or nonunion. Thus, effective drugs and targets need to be explored. In this regard, the present study aims to reveal the possible mechanism of the anti-GIOP effect of all-trans retinoic acid (ATRA).

**Methods:**

Bone morphogenetic protein 9 (BMP9)-transfected mesenchymal stem cells (MSCs) were used as an in vitro osteogenic model to deduce the relationship between ATRA and dexamethasone (DEX). The osteogenic markers runt-related transcription factor 2 (RUNX2), alkaline phosphatase (ALP), and osteopontin were detected using real-time quantitative polymerase chain reaction, Western blot, and immunofluorescent staining assay. ALP activities and matrix mineralization were evaluated using ALP staining and Alizarin Red S staining assay, respectively. The novel genes associated with ATRA and DEX were detected using RNA sequencing (RNA-seq). The binding of the protein–DNA complex was validated using chromatin immunoprecipitation (ChIP) assay. Rat GIOP models were constructed using intraperitoneal injection of dexamethasone at a dose of 1 mg/kg, while ATRA intragastric administration was applied to prevent and treat GIOP. These effects were evaluated based on the serum detection of the osteogenic markers osteocalcin and tartrate-resistant acid phosphatase 5b, histological staining, and micro-computed tomography analysis.

**Results:**

ATRA enhanced BMP9-induced ALP, RUNX2 expressions, ALP activities, and matrix mineralization in mouse embryonic fibroblasts as well as C3H10T1/2 and C2C12 cells, while a high concentration of DEX attenuated these markers. When DEX was combined with ATRA, the latter reversed DEX-inhibited ALP activities and osteogenic markers. In vivo analysis showed that ATRA reversed DEX-inhibited bone volume, bone trabecular number, and thickness. During the reversal process of ATRA, the expression of retinoic acid receptor beta (RARβ) was elevated. RARβ inhibitor Le135 partly blocked the reversal effect of ATRA. Meanwhile, RNA-seq demonstrated that serine protease inhibitor, clade A, member 3N (Serpina3n) was remarkably upregulated by DEX but downregulated when combined with ATRA. Overexpression of Serpina3n attenuated ATRA-promoted osteogenic differentiation, whereas knockdown of Serpina3n blocked DEX-inhibited osteogenic differentiation. Furthermore, ChIP assay revealed that RARβ can regulate the expression of Serpina3n.

**Conclusion:**

ATRA can reverse DEX-inhibited osteogenic differentiation both in vitro and in vivo, which may be closely related to the downregulation of DEX-promoted Serpina3n. Hence, ATRA may be viewed as a novel therapeutic agent, and Serpina3n may act as a new target for GIOP.

**Supplementary Information:**

The online version contains supplementary material available at 10.1186/s13287-021-02347-0.

## Background

Glucocorticoid-induced osteoporosis (GIOP), the most common secondary cause of osteoporosis and the most common iatrogenic disease, is caused by the high dosage and the long-term use of glucocorticoids, such as dexamethasone and prednisone [1–4]. Glucocorticoids mainly inhibit osteogenesis by suppressing the differentiation of osteoblasts and the normal functioning of osteocytes, thus inducing their apoptosis [5, 6]. On the contrary, the lifespan of osteoclasts can be prolonged and the ratio of receptor activator of nuclear factor-κΒ ligand to osteoprotegerin (RANKL/OPG) can be increased by glucocorticoids, leading to enhanced bone resorption [7–9]. Furthermore, glucocorticoids promote adipogenesis by enhancing the PPARγ and C/EBPα signaling pathways [10, 11]. GIOP patients with fractures are susceptible to delayed bone union and nonunion [12]. Hence, new anti-GIOP agents and drugs need to be explored and their underlying mechanisms need to be elucidated to prevent and treat GIOP effectively.

Bone morphogenetic protein-9 (BMP9) belongs to transforming growth factor-β (TGFβ) superfamily. As one of the most potent BMPs involved in osteogenic differentiation, BMP9 exerts its function chiefly through the BMP/Smad signaling pathway to further activate Runt-related transcription factor-2 (RUNX2) and osteogenesis [13–15]. Thus, BMP9-induced osteogenic differentiation of mesenchymal stem cells (MSCs) can be regarded as in vitro osteogenic models. Several factors and signaling pathways, such as IGF1/2 [16, 17], COX-2 [18, 19], PTEN [20], and Wnt/β-catenin, are closely related to BMP9-induced osteogenic differentiation [21, 22]. In addition, BMP9 can also activate PI3K/AKT via the noncanonical pathway to regulate cancer cell proliferation, differentiation, invasion, and metastasis [23–25].

All-trans retinoic acid (ATRA) is the major biologically active form of vitamin A. ATRA and its isomers regulate cellular growth and differentiation by binding to their nuclear receptors, such as retinoic acid receptors (RARs) and retinoid X receptors (RXRs). Both receptors have three subtypes, namely, α, β, and γ [26, 27]. ATRA binds to RARs with high affinity but binds to RXRs only when its concentration is > 10^−5^ M [28, 29]. Intracellular ATRA can be bound to cellular RA-binding proteins (CRABPs). ATRA bound to CRABPI is targeted for degradation by enzymes of the CYP26 family, whereas ATRA bound to CRABPII is further recruited into the nucleus and bound to RARs [30]. Our previous study indicated that ATRA can enhance BMP9-induced osteogenic differentiation and shift rosiglitazone-induced adipogenic differentiation to osteogenic differentiation [31, 32].

Serine protease inhibitor, clade A, member 3N (Serpina3n) is a novel inhibitory protein that was previously known as antichymotrypsin [33]. The role of Serpina3n has not yet been well-characterized. It has been reported that Serpina3n is highly expressed in brain, testis, lungs, thymus, and spleen; however, the expression of this protein is low in the bone marrow, skeletal muscle, and kidney [34]. Serpina3n is known to be involved in pathophysiological processes such as wound healing [35], pulmonary fibrosis [36], and muscle atrophy [37]. For osteogenic differentiation, Serpina3n has been reported to inhibit osteoblast phenotypes such as alkaline phosphatase (ALP), osteocalcin (OCN), and matrix mineralization [38], but little is known about the underlying mechanism. Therefore, further investigations are needed.

In this study, we combined the osteogenic inhibitory effect of DEX with the osteogenic promoting effect of ATRA. In a BMP9-induced osteogenic model, we found that the osteogenic inhibition is reversible by ATRA. We further discerned that DEX dramatically enhances the expression of Serpina3n, while in the reversal process, ATRA lowers Serpina3n expression. Furthermore, Serpina3n overexpression and knockdown confirmed that ATRA may reverse DEX-inhibited osteogenic differentiation via the attenuation of Serpina3n expression. Our findings may help in elucidating the mechanism of GIOP and in exploring novel anti-GIOP agents and drugs.

## Methods

### Cell culture and chemicals

HEK-293, C3H10T1/2, and C2C12 cells were purchased from American Type Culture Collection (ATCC, Manassas, VA, USA). The above cells were cultured in Dulbecco’s modified Eagle’s medium (DMEM), which contained 10% (v/v) fetal bovine serum (FBS), penicillin (100 U/mL), and streptomycin (100 μg/mL). Cell culturing dishes, flasks, 6-well plates, and 24-well plates were incubated at 37 °C with 5% CO_2_. DMEM, FBS, and trypsin were purchased from Gibco (Chinese branch). BMP9 (sc-514211), ALP (sc-271431), RUNX2 (sc-12488), glyceraldehyde-3-phosphate dehydrogenase (GAPDH, sc-32233) antibodies, retinoic acid receptor beta (RARβ) specific inhibitor Le135 (sc-204053), and ATRA (sc-200898) were bought from Santa Cruz Biotechnology (Chinese branch). Osteopontin (OPN, ab8448) and RARβ (ab53161) antibodies were purchased from Abcam (Chinese branch). Serpina3n (AF4709) antibody was sourced from R&D systems (Chinese branch). Dexamethasone (D4902) was obtained from Sigma-Aldrich (Chinese branch). Dylight 594 conjugated secondary antibody (A23410) was purchased from Abbkine (Chinese branch). Alkaline Phosphatase Assay Kit (C3206) and Alizarin Red S (ST1078) were bought from Beyotime (Shanghai, China).

### Construction of recombinant adenoviruses

The recombinant adenoviruses used in this study were constructed according to Ad-Easy system [39, 40]. Briefly, the coding regions of the target genes green fluorescent protein (GFP), BMP9, red fluorescent protein (RFP), and Serpina3n were cloned into the shuttle vectors as well as the Serpina3n siRNA oligo fragments. The resultant plasmids were linearized by restriction endonuclease digestion. Subsequently, these plasmids were transformed into competent AdEasier cells containing the plasmid backbone of the adenovirus. Finally, the recombinant adenovirus plasmids were digested with PacI and transfected into HEK-293 cells for 14–20 days. The recombinant adenoviruses were designated as Ad-GFP, Ad-BMP9, Ad-RFP, Ad-Serpina3n, and Ad-siSerpina3n [20, 41]. Among them, either Ad-GFP or Ad-RFP was used as a vector control to track the virus lacking target gene expression.

### Isolation of mouse embryonic fibroblasts (MEFs)

MEFs used in this study were isolated from post-coitus day 12.5 mice embryos, as described previously [42]. Briefly, each embryo was dissected and sheared using an 18-gauge syringe in the presence of 1 mL 0.25% trypsin, followed by incubation for 15 min at 37 °C with gentle shaking. Then, 10 mL DMEM containing 10% FBS was added to deactivate the trypsin. Subsequently, the dissected cells were plated in 10-cm dishes and incubated at 37 °C in the presence 5% CO_2_ for 24 h. Finally, the adherent cells were termed MEFs. All MEFs used in this study were within five passages.

### Osteogenic inductive medium (OIM) for osteogenic differentiation

OIM consisted of DEX, sodium β-glycerophosphate, and L-ascorbic acid. The three ingredients were dissolved in DMEM and filtered using a 0.2-μm filter. The obtained solution was added to DMEM containing 10% FBS. The final concentration of DEX was 10^−8^ M, sodium β-glycerophosphate was 10 mM, and L-ascorbic acid was 250 μM. For osteogenic inductive cell culturing, the cells were seeded in 24-well plates and infected with adenoviruses after adhesion. After 36 h, the medium was replaced by OIM, which was renewed once the color changed to light yellow (normally once a week) until the final Alizarin Red S staining (14 or 21 days later).

### RNA isolation, cDNA preparation, and real-time quantitative polymerase chain reaction (RTqPCR) analysis

At the corresponding timepoints, total RNA was extracted with TRIZOL reagent (Invitrogen, USA) and then purified and used for reverse transcription (RT) to generate cDNA. Later, the resultant cDNA was diluted 5–10 folds for RTqPCR assay. The expression of the housekeeping gene (GAPDH) were used to normalize the PCR results. ALP, RUNX2, RARα, RARβ, RARγ, and Serpina3n were served as the target genes. The primer sequences used in this study are presented in Table 1.

**Table 1:** Primers used in PCR assay

Gene	Assay	Forward (5′ → 3′)	Reverse (5′→ 3′)
GAPDH	PCR	ACCCAGAAGACTGTGGATGG	CACATTGGGGGTAGGAACAC
ALP	PCR	AGACCAGGTCTGCTCAGGAT	ACCCCGCTATTCCAAACAGG
RUNX2	PCR	GCCAATCCCTAAGTGTGGCT	AACAGAGAGCGAGGGGGTAT
RARα	PCR	TCTCCCTGGACATTGACCTC	GTGTCTTGCTCAGGCGTGTA
RARβ	PCR	AATGCCACCTCTCATTCAGG	GAATGTCTGCAACAGCTGGA
RARγ	PCR	AGGCAGCAGACTGACCATTT	TTCTGGTAGGTGTGCAGCAG
Serpina3n	PCR	GCTTTTCTGCACTGTGGTGG	AGGCTGTAGTCGGTGGAGAT
Serpina3n (primer 1)	ChIP	AAGGCAAGGGTGATACAGGC	TCCCTCATTGACCCCAAAGC
Serpina3n (primer 2)	ChIP	CCCCCTTTCCACATCGACTC	TGTGCTCTGTGCTCAGACTG
Serpina3n (primer 3)	ChIP	AGCCACTGAGCCTTCACTTC	TTCCTTTCTCTCAACCCGCC

### Protein harvesting and Western blot analysis

The cells were previously seeded in 6-well plates and treated with different factors according to the experiment design. At the designed timepoint, the cells in each well of the 6-well plates were washed twice with cold phosphate buffered saline (PBS, 4 °C) and lysed on ice with 300 μL of lysis buffer containing protease and phosphatase inhibitors. Subsequently, the lysed cells were transferred into 1.5-mL Eppendorf tubes, followed by the addition of 75 μL loading buffer and 15 μL β-mercaptoethanol in each tube. Then, the tubes were bathed in boiling water for 10 min. These samples were separated via sodium dodecyl sulfate polyacrylamide gel electrophoresis, and the target gels were transferred to polyvinylidene difluoride (PVDF) membranes. The membranes were bathed in 5% bovine serum albumin (BSA) for 1 h at room temperature (RT) and then incubated in the corresponding antibody solutions at RT for 1.5 h or at 4 °C overnight. The membranes were washed five times using Tris-buffered saline Tween20 (TBST) and incubated in corresponding biotin-labeled immunoglobulin G (IgG, Beyotime, Shanghai, China) for 0.5 h at RT. Next, the membranes were also washed five times using TBST and incubated in horse radish peroxidase (HRP)-labeled streptavidin (Beyotime, Shanghai, China) for 0.5 h at RT. Finally, the membranes were washed five times with TBST, and the images of the target bands on the membranes were detected using SuperSignal West Pico Chemiluminescent Substrate (SWPCS) (Thermo Scientific, IL, USA). The antibodies were diluted in 5% BSA according to the instructions (normally 1:1000), the biotin-labeled IgGs were diluted 1:3000 in TBST, and the HRP-labeled streptavidin was diluted 1:10,000 in TBST.

### Alkaline phosphatase staining assay

The cells were previously seeded in 24-well plates and treated with different factors, followed by incubation for the designated number of days. Then, the cells in the 24-well plates were washed twice with PBS and stained using the Beyotime Alkaline Phosphatase Assay Kit. Briefly, the staining solution contained ALP buffer, BCIP, and NTB in the ratio of 10 mL:33 μL:66 μL. The cells were added to 200 μL of the staining solution and were stained for 15 min in the dark. The plates were dried and photographed using a fluorescence microscope (Nikon, Japan). These results were repeated in at least three independent experiments.

### Alizarin Red S staining assay

The cells seeded in the 24-well plates were treated with different factors and incubated for 14 or 21 days. Subsequently, the cells were washed twice with PBS and fixed with 4% paraformaldehyde for 10 min. Then, the cells were washed gently using pH 4.2 PBS and were stained with 0.4% Alizarin Red S solution. Finally, the plates were scanned and the images were captured using a fluorescence microscope (Nikon, Japan). For quantification, the mineralized matrix was bathed in 10% acetic acid and the absorbance at 405 nm was measured using a microplate reader as previously described [18, 41]. These results were repeated in at least three independent experiments.

### Immunofluorescence staining assay

The cells were firstly seeded in 48-well plates and treated with the corresponding factors, followed by incubation at 37 °C for 24 h. Then cells were washed twice with PBS and fixed with 4% paraformaldehyde for 10 min. Subsequently, they were bathed in 0.3% TritonX-100 (Solarbio, Beijing, China) to increase the permeability of the cell membrane, followed by three washes with PBS. Then, they were blocked with goat serum (Beyotime, Shanghai, China) at RT for 1 h and later bathed in the target antibodies at 4 °C overnight. The antibodies were recycled on the next day, and the cells were washed thrice with PBS, followed by incubation entirely in the dark with Dylight 594 conjugated secondary antibody at 37 °C for 1 h. Then, the cells were washed thrice with PBS and stained with 4,6-diamino-2-phenyl indole (DAPI) for 5 min. Finally, they were washed thrice with PBS and bathed in 80% glycerin for immunofluorescent image capture. The dilution rates were 1:200 for antibodies, 1:50 for the secondary antibody, and 1:10,000 for DAPI. These results were repeated in at least three independent experiments.

### RNA sequencing assay

The MEFs in the seven dishes were divided into seven groups: GFP + DMSO, GFP + DEX, GFP + ATRA, BMP9 + DMSO, BMP9 + DEX, BMP9 + ATRA, and BMP9 + DEX + ATRA. The cells were treated with the corresponding factors and cultured for 36 h. For the first 12 h, they were treated only with Ad-GFP or Ad-BMP9. DEX or ATRA was added 12 h after adenovirus infection. The concentration of DEX was 10^−6^ M and that of ATRA was 1 μM. Then, the MEFs were washed twice with PBS and extracted on ice using TRIZOL agent (Invitrogen). The RNA-seq experiments were performed by Novogene Biotech Co. Ltd. (Beijing, China). Briefly, the sequencing libraries were generated using NEBNext UltraTM RNA Library Prep Kit for Illumina (NEB, USA) according to the manufacturer’s instructions. The library quality was assessed using the Agilent Bioanalyzer 2100 system. Subsequently, the gene expression profiles of the seven groups were analyzed using the Illumina Hiseq platform, followed by data analysis of the differentially expressed genes.

### Chromatin immunoprecipitation assay

ChIP was performed using the ChIP Assay Kit (Beyotime, Beijing, China) as previously described [43]. Briefly, the MEFs in the dishes were crosslinked with 1% formaldehyde (final concentration) for 10 min. The reactions were terminated by the addition of glycine to a final concentration of 125 mM for 5 min. Subsequently, the cells were washed with cold PBS and lysed with SDS lysis buffer containing protease inhibitors. Then, the lysate was sonicated to generate 400–800-bp sequences, incubated with 5 M NaCl at 65 °C for 4 h, extracted with phenol–chloroform, and precipitated with ethanol. One third of the lysate was stored at − 80 °C to be used for subsequent PCR analysis, and the remaining two thirds were subjected to immunoprecipitation using RARβ antibody or goat IgG. Finally, the precipitated protein–DNA complex was pulled down with Protein A + G Agarose/Salmon Sperm DNA, incubated with 5 M NaCl, and extracted with phenol–chloroform for further PCR analysis. Goat IgG was employed as the negative control. Primers of Serpina3n used in the ChIP assay are listed in Table 1.

### Construction of GIOP rat model and ATRA treatment

All animal experiments were approved by the Ethics Committee of Chongqing Medical University (CQMU). Forty 8-week-old male Sprague-Dawley (SD) rats weighing 200 ± 10 g were obtained from the Animal Center of CQMU and were divided into eight groups as follows (*n* = 5): control group, GIOP group (DEX), DEX + prevention with low-dose ATRA group (DEX + LP), DEX + prevention with medium dose ATRA group (DEX + MP), DEX + prevention with high-dose ATRA group (DEX + HP), DEX + treatment with low-dose ATRA group (DEX + LT), DEX + treatment with medium-dose ATRA group (DEX + MT), and DEX + treatment with high-dose ATRA group (DEX + HT). After 1 week of acclimation, the rats were treated with the corresponding agents in the following manner: the rats in the DEX-mediated groups (except for the control group) were intraperitoneally injected with DEX at a dose of 1 mg/kg (twice a week) for 8 weeks, and the rats in the control group were intraperitoneally injected with the same volume of normal saline for 8 weeks. ATRA was ground with sodium carboxymethyl cellulose and dissolved in water (in the high dose group, ATRA might not have been completely dissolved). In the ATRA prevention group, the rats were intragastrically administrated with ATRA for 8 weeks: 0.2 mg/kg/day for the LP group, 1.0 mg/kg/day for the MP group, and 5.0 mg/kg/day for the HP group. In the ATRA treatment group, the rats were intragastrically administrated with ATRA for 4 weeks after DEX injection: 0.2 mg/kg/day for the LT group, 1.0 mg/kg/day for the MT group, and 5.0 mg/kg/day for the HT group. After 8 weeks, the rats were sacrificed and the blood and lumbar vertebrae of each group were collected for further serological and histological evaluations and micro-computed tomography (μCT) analysis.

### Estimation of the serum concentration of bone metabolism-related markers

The collected tubes of blood were coagulated for 30 min and centrifuged at 4 °C for 10 min, and the supernatants (sera) were retrieved. Serum ALP (405 nm), calcium (660 nm), and phosphorus (340 nm) concentrations were estimated by using the corresponding kits. The absorbance values were measured using a microplate reader, and the optical density (OD) values were converted to concentration. For serum OCN and tartrate-resistant acid phosphatase 5b (TRACP-5b), enzyme-linked immunosorbent assay (ELISA) was employed for concentration detection via the corresponding kit. Briefly, the obtained OD values were calibrated using the standard sample and were converted to the concentrations according to the formula derived from the standard sample.

### Histological evaluation and staining

The obtained lumbar vertebrae of the rats were fixed with 4% paraformaldehyde for 1 week, decalcified with EDTA solution (pH 7.2) for 4 weeks, dehydrated with ethanol, and embedded with paraffin. Subsequently, the paraffin-coated masses were sliced and subjected to hematoxylin–eosin (H&E) staining and Masson’s trichrome staining after deparaffinization and rehydration.

### Micro-computed tomographic analysis and 3D reconstruction

All harvested lumbar vertebral samples were scanned with μCT (VivaCT 40, SCANCO Medical AG, Switzerland). The transverse plane, 3D reconstructions, and ROI analysis, such as the ratio of bone volume to total volume (BV/TV), trabecular number (Tb.N), trabecular thickness (Tb.Th), and trabecular separation (Tb.Sp), were performed using the software linked to the scanner (μCT 516.1).

### NIH ImageJ for quantification of protein bands and ALP activities

**Fig. 1:**
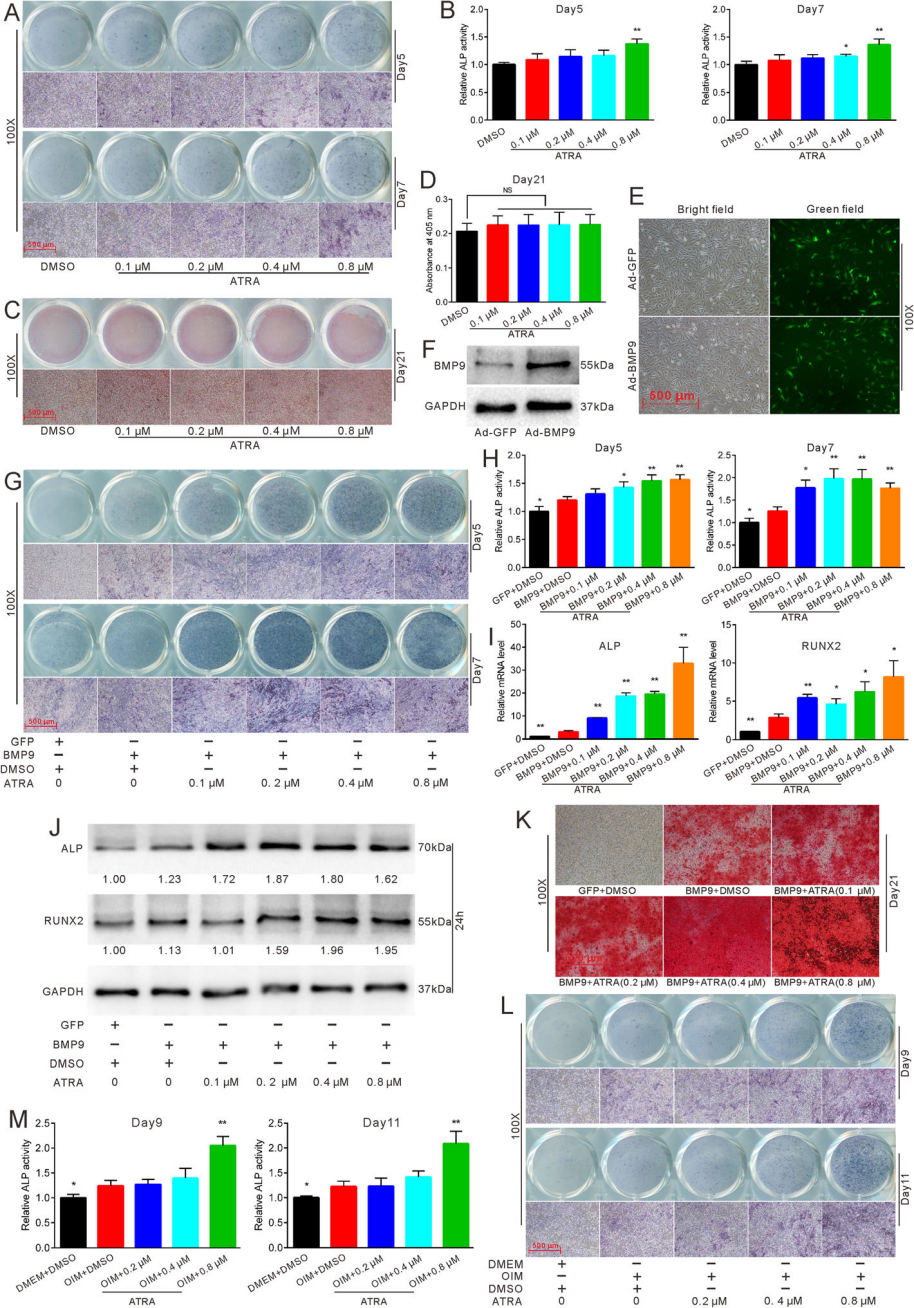
Effects of ATRA on osteogenic differentiation in MEFs. **a** ALP staining showed the effect of different concentrations of ATRA on ALP activities in MEFs after 5 and 7 days. **b** Quantification of ALP staining demonstrated the effect of different concentrations of ATRA on ALP activities in MEFs after 5 and 7 days, *p < 0.05 vs DMSO, **p < 0.01 vs DMSO. **c** Alizarin Red S staining showed the effect of different concentrations of ATRA on matrix mineralization in MEFs after 21 days. **d** Quantification of matrix mineralization in MEFs after 21 days, *p < 0.05 vs DMSO, **p < 0.01 vs DMSO. **e** MEFs infected with adenovirus-mediated Ad-GFP and Ad-BMP9 after 24 h. **f** Western blot demonstrated the protein expression of BMP9 in MEFs 24 h after Ad-GFP and Ad-BMP9 infection. **g** ALP staining showed the effect of different concentrations of ATRA on BMP9-induced ALP activities in MEFs after 5 and 7 days. **h** Quantification of ALP staining showed the effect of different concentrations of ATRA on BMP9-induced ALP activities in MEFs after 5 and 7 days, *p < 0.05 vs BMP9 + DMSO, **p < 0.01 vs BMP9 + DMSO. **i** RTqPCR showed the effect of different concentrations of ATRA on BMP9-induced osteogenic markers after 24 h, *p < 0.05 vs BMP9 + DMSO, **p < 0.01 vs BMP9 + DMSO. **j** Western blot results showed the effect of different concentrations of ATRA on BMP9-induced osteogenic markers after 24 h (numbers between the bands were the ratios of target bands to GAPDH using ImageJ). **k** Microscopic view of Alizarin Red S staining showed the effect of different concentrations of ATRA on BMP9-induced matrix mineralization in MEFs after 21 days. **l** ALP staining showed the effect of different concentrations of ATRA on OIM-induced ALP activities in MEFs after 9 and 11 days. **m** Quantification of ALP staining demonstrated the effect of different concentrations of ATRA on OIM-induced ALP activities in MEFs after 9 and 11 days, *p < 0.05 vs OIM + DMSO, **p < 0.01 vs OIM + DMSO. GFP, green fluorescent protein; DMSO, dimethyl sulfoxide; DMEM, Dulbecco’s modified Eagle’s medium; OIM, osteogenic inductive medium

The software NIH ImageJ (http://rsbweb.nih.gov/ij/download.html) was used to quantify the protein bands and the ALP staining images. For the protein bands in Fig. 1, the ratios of the target protein to GAPDH normalized to the control (GFP + DMSO) are shown beneath the target bands. For the protein bands in other figures, the triplicated results were quantified, normalized to their control, and subjected to further statistical analysis. For ALP quantification, the images were black-white colored and color inverted, and each well was measured for further statistical analysis.

### Statistical analysis

For statistical analysis, GraphPad Prism 6 software was used to analyze the standard deviations and the differences between two groups via two-tailed Student’s *t* test. *p* values < 0.05 were considered significant, and *p* values < 0.01 were considered remarkably significant. For all quantitative analysis, the experiments were carried out in triplicates, and the results were repeated in at least three independent experiments.

## Results

### Effects of ATRA on BMP9-induced and OIM-induced osteogenic differentiation of MEFs

Although the effect of ATRA on osteoblasts and osteocytes remains controversial, several research findings have demonstrated its positive effect on the osteogenic differentiation of MSCs. Therefore, we firstly checked the effect of ATRA on the osteogenic differentiation of MEFs. ALP staining assay showed that ATRA can induce ALP activities in a concentration-dependent manner (Fig. 1a, b). Next, we evaluated the effect of ATRA on matrix mineralization. Alizarin Res S staining and quantification established that ATRA alone cannot induce matrix mineralization (Fig. S1A-B and Fig. 1c, d). Subsequently, we utilized adenovirus-mediated BMP9 and validated its function (Fig. 1e, f). BMP9 was capable of solely inducing ALP activities; in addition, ATRA enhanced the ALP activities induced by BMP9 in a statistically significant manner (Fig. 1g, h). This effect was confirmed in C3H10T1/2 and C2C12 cells (Fig. S1C-H). RTqPCR and Western blot results of the osteogenic markers ALP and RUNX2 were consistent with those of ALP staining (Fig. 1i, j). Furthermore, Alizarin Red S staining confirmed that ATRA can enhance BMP9-induced osteogenic differentiation (Fig. 1k and Fig. S1I-J). Finally, we replaced IOM with BMP9, and ATRA was found to promote OIM-induced ALP activities as well (Fig. 1l, m). These results suggest that ATRA can induce ALP activities but not matrix mineralization on their own. Meanwhile, ATRA can enhance both BMP9-induced and OIM-induced osteogenic differentiation.

### Effects of DEX on BMP9-induced and OIM-induced osteogenic differentiation of MEFs

**Fig. 2:**
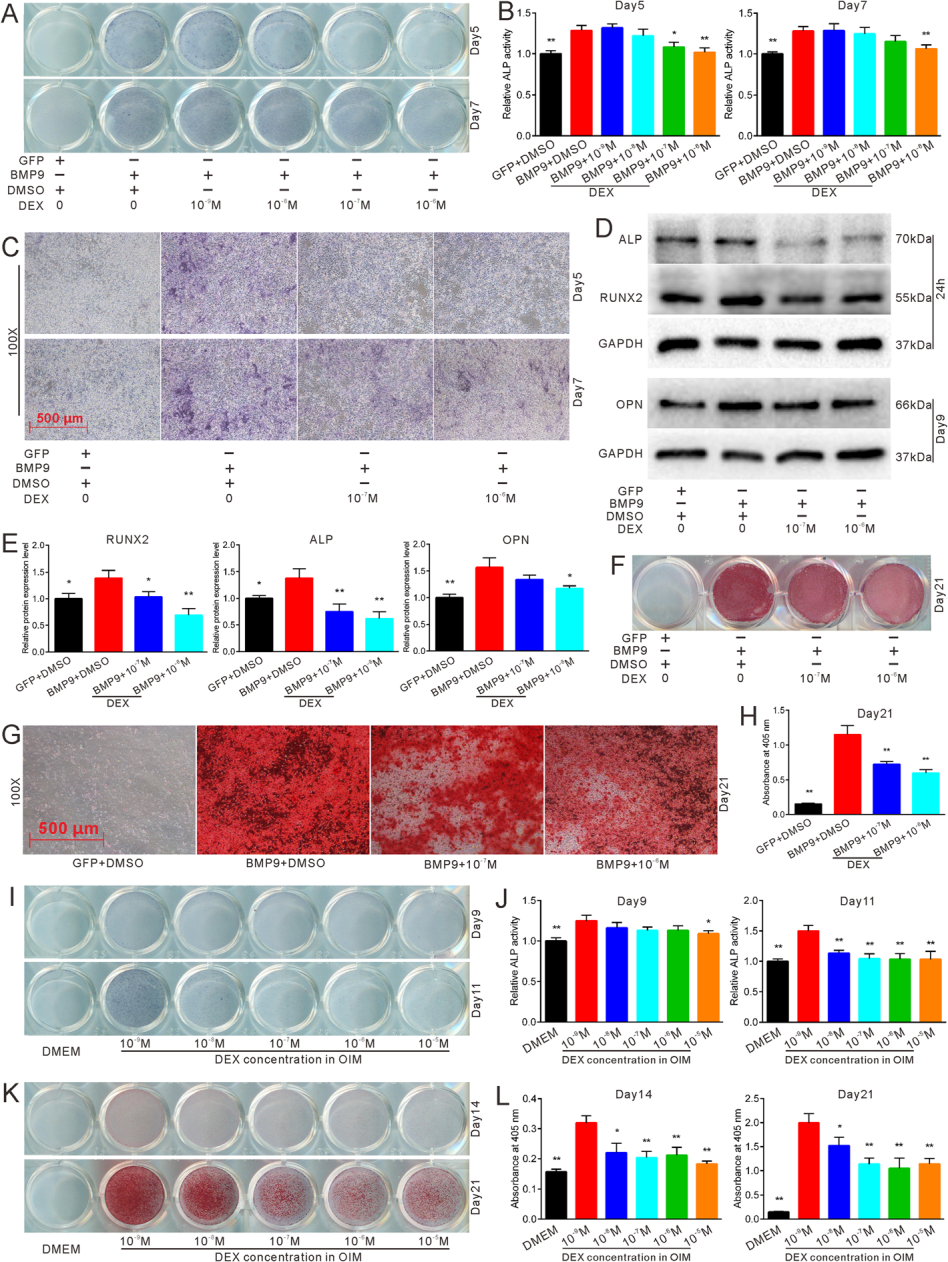
Effects of DEX on osteogenic differentiation in MEFs. **a** ALP staining demonstrated the effect of different concentrations of DEX on BMP9-induced ALP activities in MEFs after 5 and 7 days. **b** Quantification of ALP staining demonstrated the effect of different concentrations of DEX on BMP9-induced ALP activities in MEFs after 5 and 7 days, *p < 0.05 vs BMP9 + DMSO, **p < 0.01 vs BMP9 + DMSO. **c** Microscopic view of ALP staining showed the effect of different concentrations of DEX on BMP9-induced ALP activities in MEFs after 5 and 7 days. **d** Western blot results showed the effect of different concentrations of DEX on BMP9-induced osteogenic markers. **e** Quantification of western blot demonstrated the effect of different concentrations of DEX on BMP9-induced osteogenic marker ALP, RUNX2, and OPN, *p < 0.05 vs BMP9 + DMSO, **p < 0.01 vs BMP9 + DMSO. **f** Alizarin Red S staining showed the effect of different concentrations of DEX on matrix mineralization in MEFs after 21 days. **g** Microscopic view of Alizarin Red S staining demonstrated the effect of different concentrations of DEX on BMP9-induced matrix mineralization. **h** Quantification of matrix mineralization in MEFs, *p < 0.05 vs BMP9 + DMSO, **p < 0.01 vs BMP9 + DMSO. **i** ALP staining demonstrated the effect of different concentrations of OIM on ALP activities in MEFs after 9 and 11 days. **j** Quantification of ALP staining showed the effect of different concentrations of OIM on ALP activities, *p < 0.05 vs OIM with 10^−9^ M DEX, **p < 0.01 vs OIM with 10^-9^ M DEX. **k** Alizarin Red S staining demonstrated the effect of different concentrations of OIM on matrix mineralization in MEFs after 14 and 21 days. **l** Quantification of Alizarin Red S staining showed the effect of different concentrations of OIM on matrix mineralization, *p < 0.05 vs OIM with 10^−9^ M DEX, **p < 0.01 vs OIM with 10^−9^ M DEX. GFP, green fluorescent protein; DMSO, dimethyl sulfoxide; DEX, dexamethasone; OPN, osteopontin, DMEM, Dulbecco’s modified Eagle’s medium; OIM, osteogenic inductive medium

Based on the knowledge that a high concentration of DEX inhibits osteogenesis, we used different concentrations of DEX to explore its effects on BMP9-induced and OIM-induced osteogenic differentiation. At a concentration of 10^−9^ M and 10^−8^ M, DEX showed no obvious inhibition of BMP9-induced ALP activities; however, at 10^−6^ M, it resulted in the significant inhibition (Fig. 2a–c and Fig. S2A-F) of different MSCs. Western blot analysis and the results of quantification revealed that high concentrations of DEX (10^−7^ M and 10^−6^ M) can attenuate the protein expressions of the early osteogenic markers RUNX2 and ALP and the late osteogenic marker OPN induced by BMP9 (Fig. 2d, e). Alizarin Res S staining and quantification also demonstrated that high concentrations of DEX inhibited BMP9-induced matrix mineralization (Fig. 2f–h). Given that DEX is an ingredient of OIM, we altered its concentration in OIM instead of adding different concentrations of it to OIM-induced MEFs. ALP and Alizarin Red S staining and the quantifications demonstrated that high concentrations of DEX reduced ALP activities and matrix mineralization (Fig. 2i–l). These data imply that high concentrations of DEX can inhibit BMP9-induced and OIM-induced osteogenic differentiation.

### Effects of ATRA on DEX-inhibited osteogenic differentiation

**Fig. 3:**
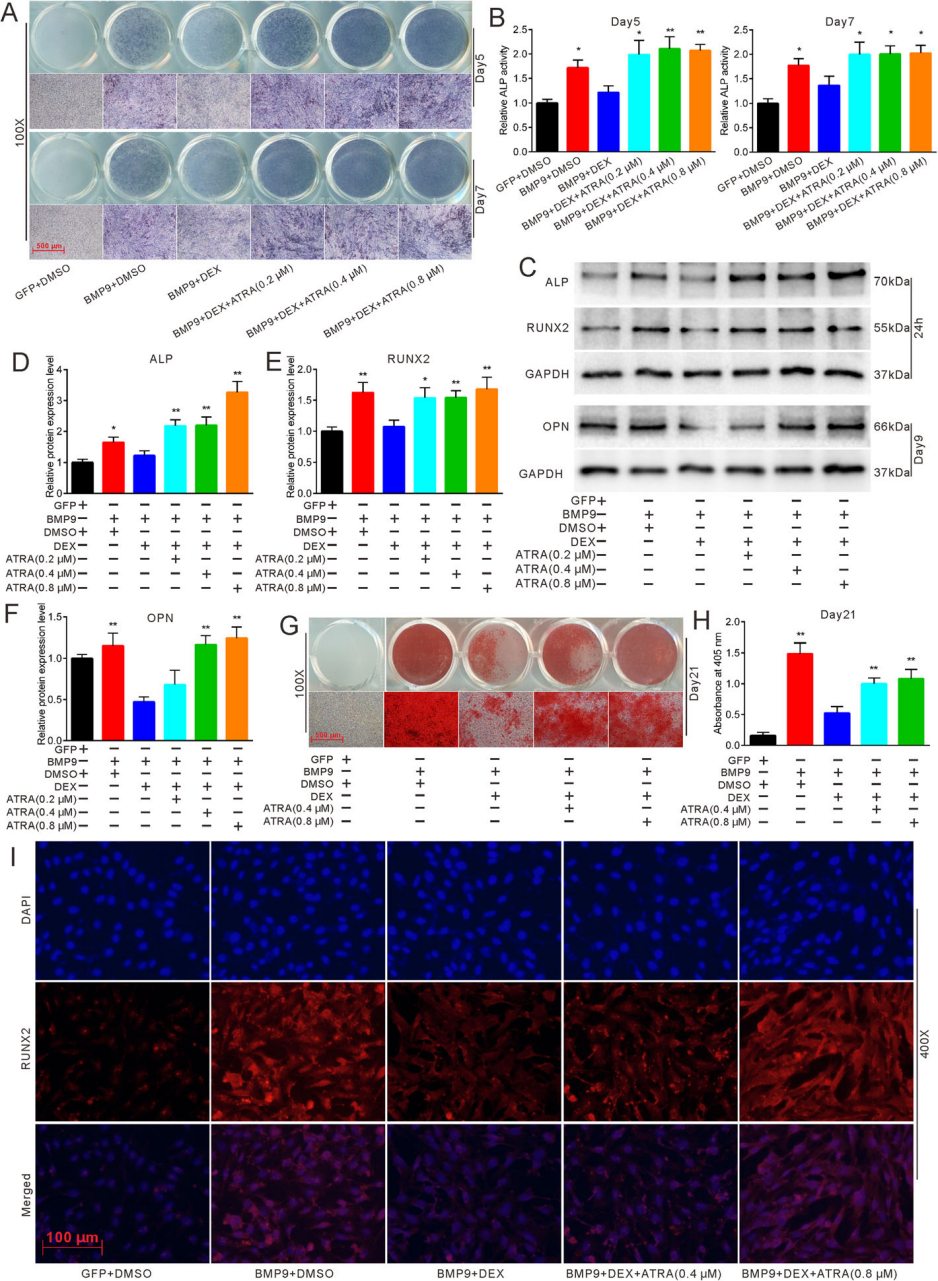
Effects of ATRA on DEX-inhibited osteogenic differentiation. **a** ALP staining demonstrated the effect of different concentrations of ATRA on DEX-inhibited ALP activities in MEFs after 5 and 7 days. **b** Quantification of ALP staining showed the effect of different concentrations of ATRA on DEX-inhibited ALP activities, *p < 0.05 vs BMP9 + DEX, **p < 0.01 vs BMP9 + DEX. **c** Western blot results demonstrated the effect of different concentrations of ATRA on DEX-inhibited protein expression of osteogenic markers. **d**–**f** Quantification of western blot showed the effect of different concentrations of ATRA on DEX-inhibited osteogenic marker ALP, RUNX2, and OPN, *p < 0.05 vs BMP9 + DEX, **p < 0.01 vs BMP9 + DEX. **g** Alizarin Red S staining showed the effect of different concentrations of ATRA on DEX-inhibited matrix mineralization in MEFs after 21 days. **h** Quantification of matrix mineralization demonstrated the effect of ATRA on DEX-inhibited matrix mineralization in MEFs, *p < 0.05 vs BMP9 + DEX, **p < 0.01 vs BMP9 + DEX. **i** Immunofluorescent staining showed the effect of different concentrations of ATRA on DEX-inhibited RUNX2 expression after 24 h. GFP, green fluorescent protein; DMSO, dimethyl sulfoxide; DEX, dexamethasone; OPN, osteopontin

Next, we aimed to study the combined effect of ATRA and DEX on BMP9-induced osteogenic differentiation. To accurately evaluate the effect of ATRA, 10^−6^ M, which is the most effective concentration of DEX, was chosen for the following experiments. ALP staining indicated that ATRA reversed DEX-inhibited ALP activities and that their combined effects were stronger than those of the BMP9 alone group (Fig. 3a, b). The osteogenic markers ALP, RUNX2, and OPN (Fig. 3c–f) and matrix mineralization (Fig. 3g, h) inhibited by DEX were also reversed by ATRA. Subsequently, we utilized immunofluorescence staining, and the results revealed that the fluorescence of RUNX2 was stronger in the DEX and ATRA group when compared with the DEX alone group (Fig. 3i). We then employed C3H10T1/2 cells for further experiments. ALP staining illustrated that ATRA can reverse the inhibition of ALP activities caused by different concentrations (10^−9^–10^−6^ M) of DEX (Fig. S3A-E). These results allude that ATRA can reverse DEX-inhibited osteogenic differentiation in different MSCs.

### Effects of ATRA on bone metabolism in the GIOP rat model

**Fig. 4:**
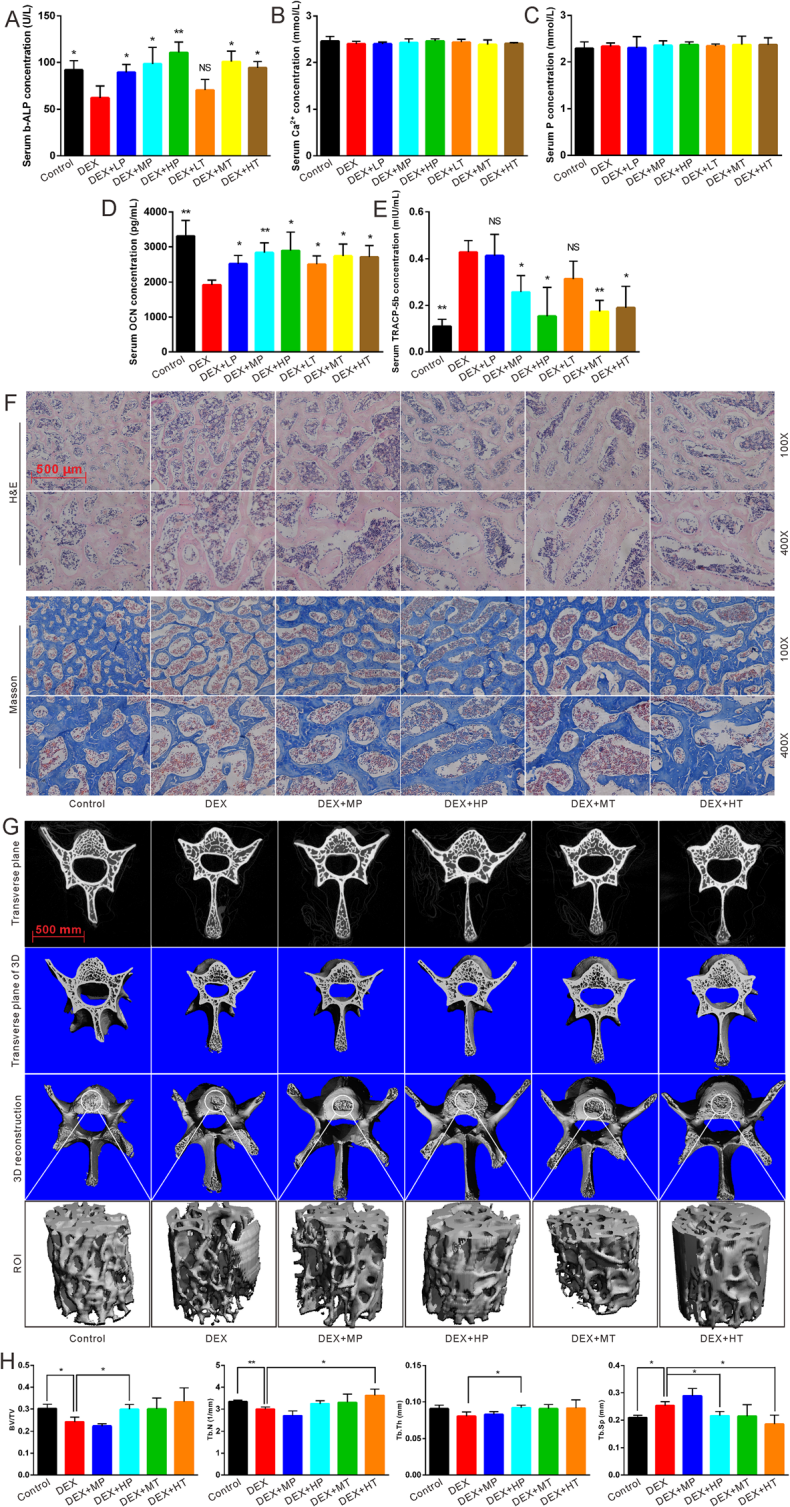
Effects of ATRA on GIOP rat model in vivo. **a–e** Serum concentrations of ALP, calcium, phosphorus, OCN, and TRACP-5b in different groups, *p < 0.05 vs DEX, **p < 0.01 vs DEX. **f** H&E and Masson’s trichrome staining showed the effect of ATRA on DEX-induced GIOP rats. **g** μCT and three-dimensional reconstruction of vertebrae demonstrated the effect of ATRA on DEX-induced GIOP rats. **h** ROI analysis of vertebrae showed the effect of ATRA on DEX-induced GIOP rats, *p < 0.05, **p < 0.01. OCN, osteocalcin; TRACP-5b, tartrate-resistant acid phosphatase 5b; ROI, region of interest; BV/TV, ratio of bone volume to total volume; Tb.N, trabecular number; Tb.Th, trabecular thickness; Tb.Sp, trabecular separation; LP: prevention with low concentration of ATRA; MP: prevention with medium concentration of ATRA; HP: prevention with high concentration of ATRA; LT: treatment with low concentration of ATRA; MT: treatment with medium concentration of ATRA; HT: treatment with high concentration of ATRA

To further investigate the effects of ATRA on DEX-inhibited osteogenesis, we constructed a GIOP rat model for prevention and treatment using ATRA. Estimation of the serum concentrations of ALP, calcium, and phosphorus revealed that DEX lowered the serum concentration of ALP. Both prevention and treatment using ATRA reversed DEX-lowered ALP concentration (Fig. 4a). However, both DEX and ATRA showed no effect on the serum concentrations of calcium and phosphorus (Fig. 4b, c). ELISA results demonstrated that the serum concentration of the osteoblastic marker OCN downregulated by DEX was reversed by ATRA in the prevention and treatment groups (Fig. 4d). In contrast, the serum osteoclastic marker TRACP-5b was enhanced by DEX but attenuated when combined with ATRA (Fig. 4e). H&E and Masson’s trichrome staining showed that the number and thickness of the trabecular bone were dramatically decreased by DEX and were partly reversed by the ATRA combination (Fig. 4f), irrespective of whether the vertebrae were from the prevention group or the treatment group. Furthermore, μCT scan, 3D reconstruction, and ROI analysis revealed that the bone trabecular changes in each group were in accordance with the histological evaluations (Fig. 4g, h). These data suggest that ATRA can reverse GIOP in the rat model, both in the prevention and treatment groups.

### Effects of RARβ on the reversal process of ATRA with regard to DEX-inhibited osteogenic differentiation of MEFs

**Fig. 5:**
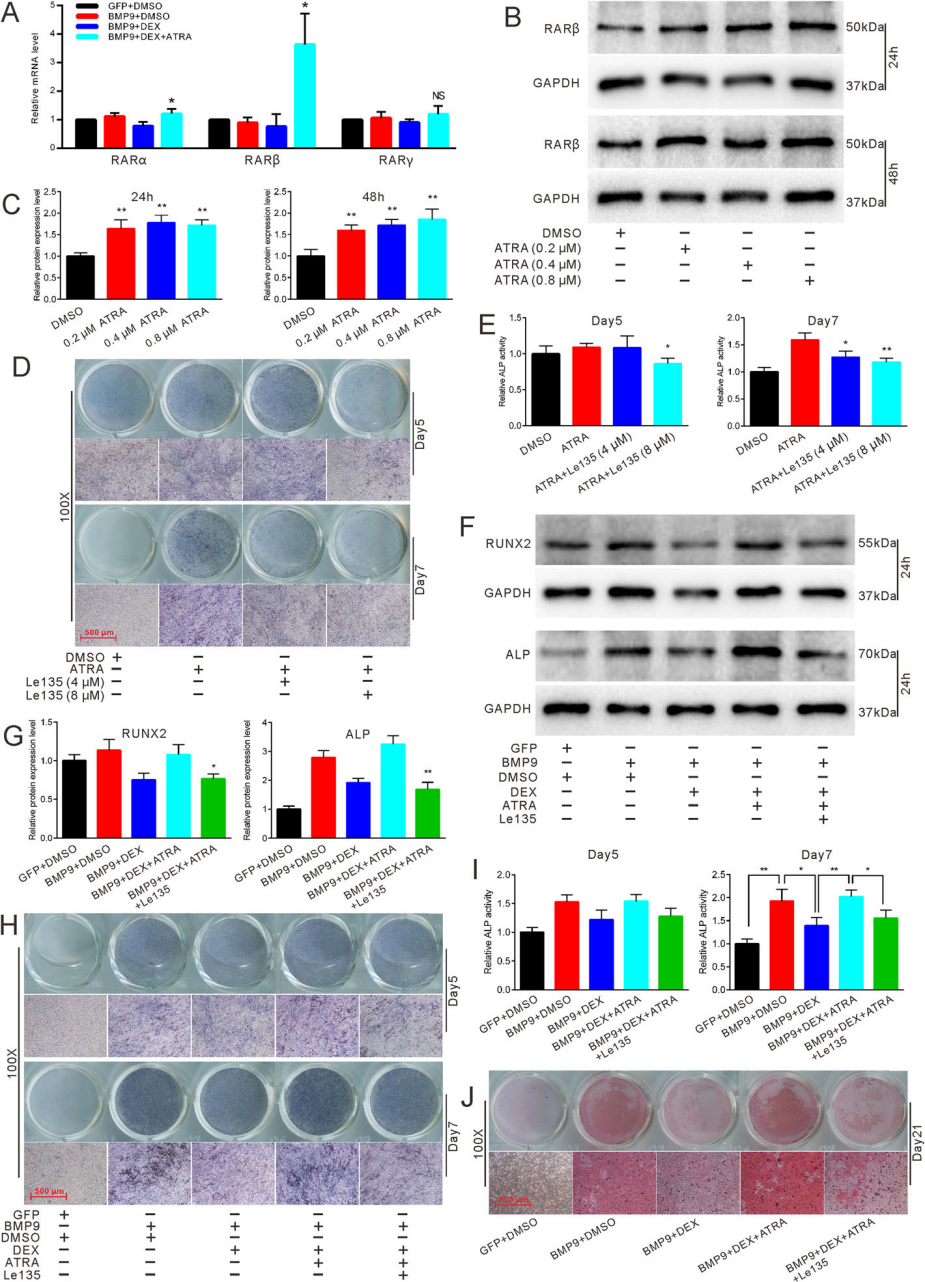
Effects of RARβ in the reversal process of ATRA in DEX-inhibited osteogenic differentiation. **a** RTqPCR results showed the effect of ATRA on mRNA expression of RARβ in DEX-inhibited osteogenic differentiation in MEFs. **b** Western blot results showed the effect of different concentrations of ATRA on protein expression of RARβ in MEFs. **c** Quantification of western blot showed the effect of ATRA on the expression of RARβ in MEFs, *p < 0.05 vs DMSO, **p < 0.01 vs DMSO. **d** ALP staining demonstrated the effect of Le135 on ATRA-induced ALP activities in MEFs after 5 and 7 days. **e** Quantification of ALP staining showed the effect of Le135 on ATRA-induced ALP activities, *p < 0.05 vs ATRA, **p < 0.01 vs ATRA. **f** Western blot results showed the effect of Le135 on ATRA-reversed protein expression of osteogenic markers. **g** Quantification of western blot showed the effect of Le135 on ATRA-reversed osteogenic marker RUNX2 and ALP, *p < 0.05 vs BMP9 + DEX + ATRA, **p < 0.01 vs BMP9 + DEX + ATRA. **h** ALP staining demonstrated the effect of Le135 on ATRA-reversed ALP activities in MEFs after 5 and 7 days. **i** Quantification of ALP staining showed the effect of Le135 on ATRA-reversed ALP activities, *p < 0.05 vs BMP9 + DEX + ATRA, **p < 0.01 vs BMP9 + DEX + ATRA. **j** Alizarin Red S staining showed the effect of Le135 on ATRA-reversed matrix mineralization in MEFs after 21 days. GFP, green fluorescent protein; DMSO, dimethyl sulfoxide; DEX, dexamethasone; RARβ, retinoic acid receptor β; Le135, specific inhibitor of RARβ

Based on the knowledge that low concentrations of ATRA only functionally bind to RARs, we assessed the three types of RARs (RARα, RARβ, and RARγ). We found that RARβ was obviously upregulated by ATRA (Fig. 5a). Next, we checked the expression of RARβ using different concentrations of ATRA. Western blot results and quantifications showed that RARβ was upregulated by ATRA in a concentration-dependent manner (Fig. 5b, c). We exploited Le135, a specific RARβ inhibitor, and found that ATRA-induced ALP activities can be attenuated by it (Fig. 5d, e). In addition, Le135 also attenuated ATRA-reversed protein expressions of the osteogenic markers RUNX2 and ALP (Fig. 5f, g), ALP activities (Fig. 5h, i), and matrix mineralization (Fig. 5j). These results suggest that ATRA may reverse DEX-inhibited osteogenic differentiation by binding to RARβ and upregulating its expression.

### Effects of DEX and ATRA on the expression of Serpina3n in MEFs

**Fig. 6:**
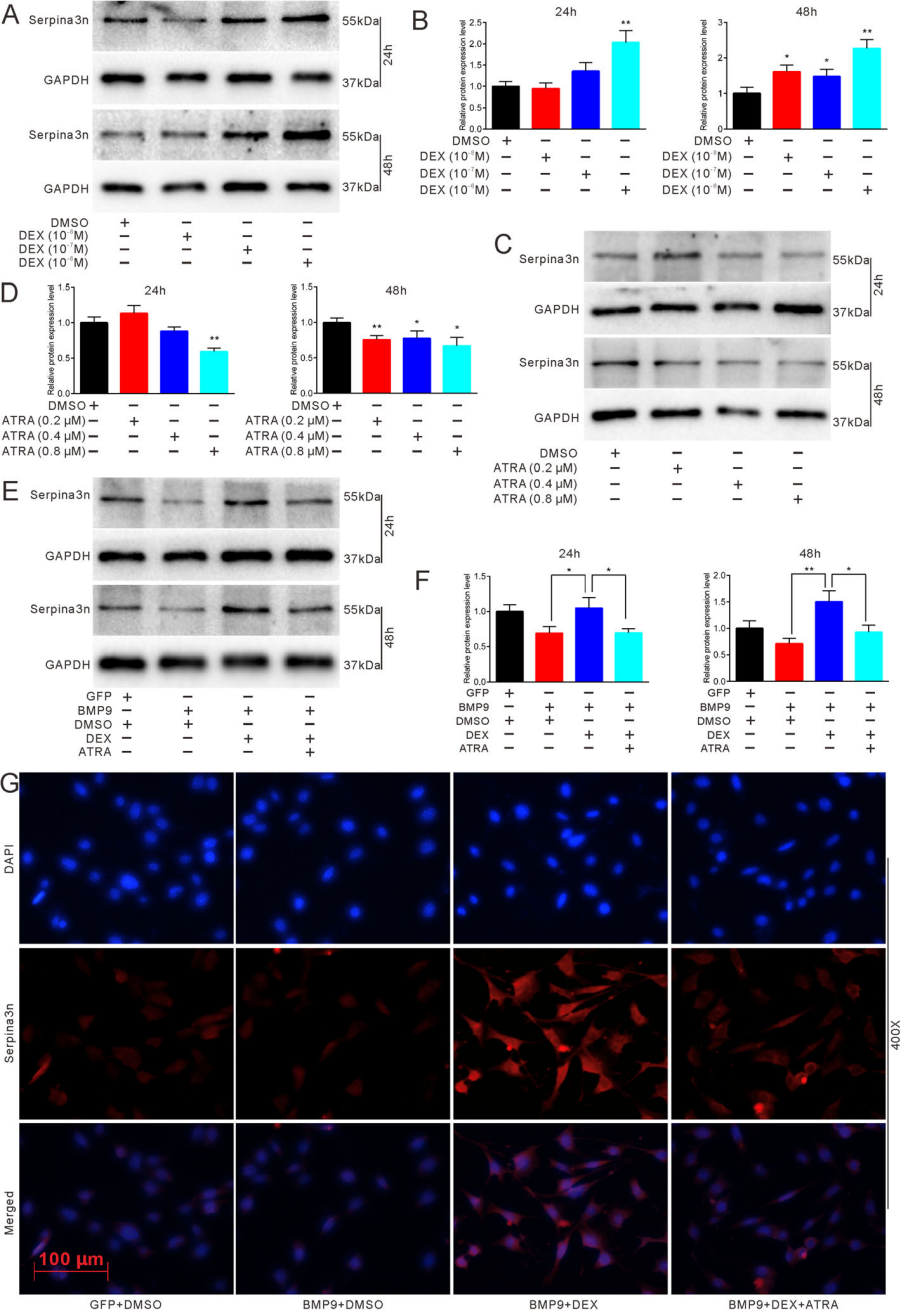
Effects of ATRA and DEX on the expression of Serpina3n. **a** Western blot results showed the effect of different concentrations of DEX on protein expression of Serpina3n in MEFs. **b** Quantification of western blot showed the effect of different concentrations of DEX on the expression of Serpina3n in MEFs, *p < 0.05 vs DMSO, **p < 0.01 vs DMSO. **c** Western blot results showed the effect of different concentrations of ATRA on protein expression of Serpina3n in MEFs. **d** Quantification of western blot showed the effect of different concentrations of ATRA on the expression of Serpina3n in MEFs, *p < 0.05 vs DMSO, **p < 0.01 vs DMSO. **e** Western blot results showed the effect of ATRA and DEX on the expression of Serpina3n in BMP9-induced MEFs. **f** Quantification of western blot showed the effect of ATRA and DEX on the expression of Serpina3n in BMP9-induced MEFs, *p < 0.05, **p < 0.01. **g** Immunofluorescent staining demonstrated the effect of ATRA and DEX on Serpina3n expression in BMP9-induced MEFs. GFP, green fluorescent protein; DMSO, dimethyl sulfoxide; DEX, dexamethasone

To further explore the reversal mechanism of ATRA, we performed RNA-seq and found the novel gene Serpina3n, which has not been well-characterized so far. Western blot results and quantifications demonstrated that DEX can upregulate the protein expression of Serpina3n in a concentration-dependent manner (Fig. 6a, b). On the contrary, ATRA lowered the expression of Serpina3n (Fig. 6c, d). Next, we found that the expression of Serpina3n was also promoted by DEX and inhibited by ATRA through the mediation of BMP9 (Fig. 6e, f). Moreover, the results of immunofluorescence staining were consistent with those of Western blot analysis (Fig. 6g). These data imply that Serpina3n can be regulated by DEX and ATRA. Meanwhile, Serpina3n may be involved in the reversal caused by ATRA with regard to DEX-inhibited osteogenesis.

### Effects of Serpina3n on the reversal process of ATRA with regard to DEX-inhibited osteogenesis

**Fig. 7:**
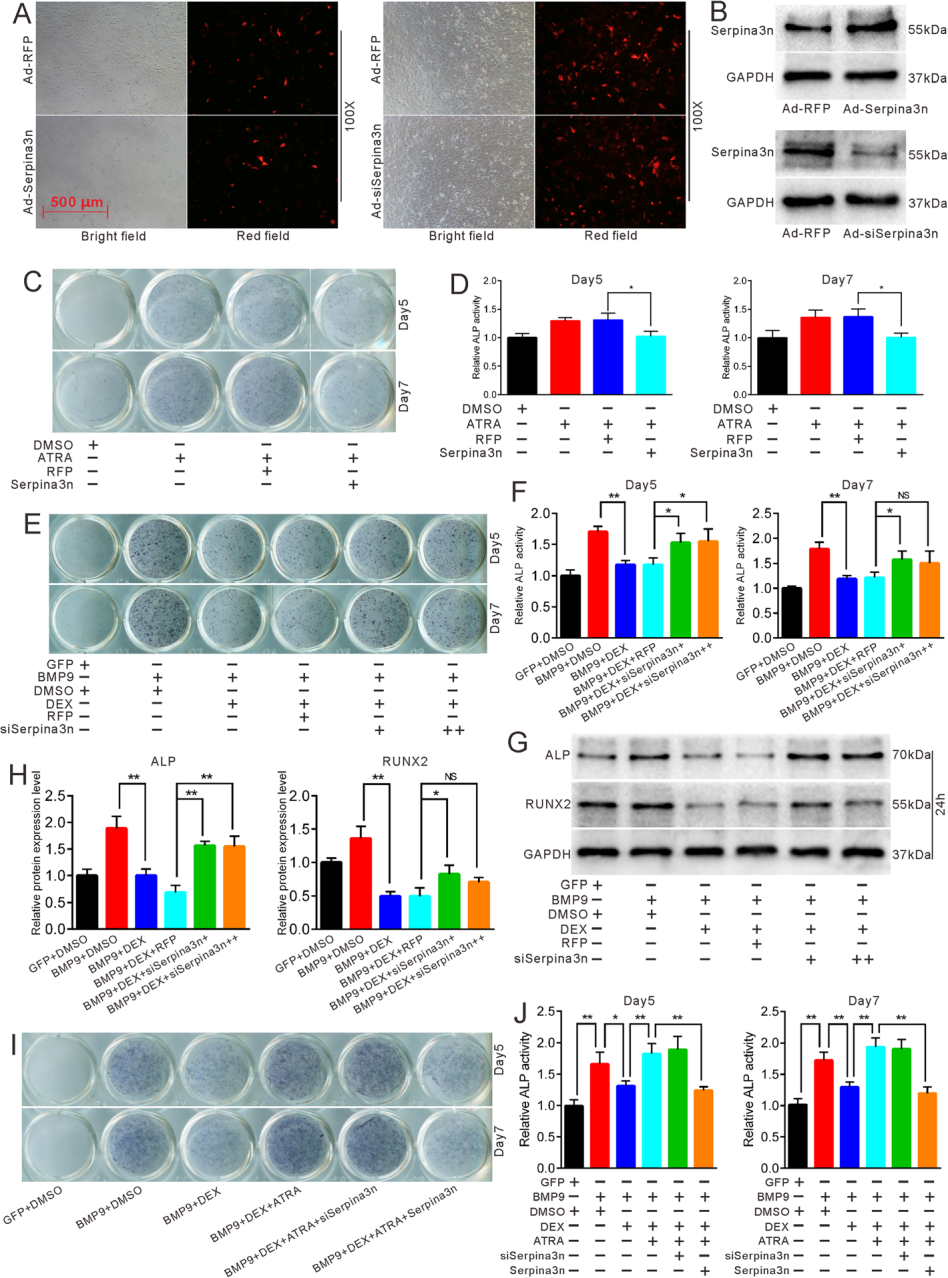
Effects of Serpina3n overexpression and knockdown on the reversal process of ATRA in DEX-inhibited osteogenic differentiation. **a** MEFs infected with adenovirus-mediated Ad-RFP, Ad-siSerpina3n, and Ad-Serpina3n after 24 h. **b** Western blot showed the protein expression of Serpina3n in MEFs 24 h after Ad-RFP, Ad-siSerpina3n and Ad-Serpina3n infection. **c** ALP staining demonstrated the effect of overexpression of Serpina3n on ATRA-induced ALP activities in MEFs after 5 and 7 days. **d** Quantification of ALP staining showed the effect of overexpression of Serpina3n on ATRA-induced ALP activities, *p < 0.05, **p < 0.01. **e** ALP staining showed the effect of knockdown of Serpina3n on DEX-inhibited ALP activities induced by BMP9. **f** Quantification of ALP staining showed the effect of knockdown of Serpina3n on DEX-inhibited ALP activities, *p < 0.05, **p < 0.01. **g** Western blot results showed the effect of knockdown of Serpina3n on DEX-inhibited osteogenic marker ALP and RUNX2 induced by BMP9 in MEFs. **h** Quantification of western blot demonstrated the effect of knockdown of Serpina3n on DEX-inhibited osteogenic markers in MEFs, *p < 0.05, **p < 0.01. **i** ALP staining showed the effect of Serpina3n overexpression and knockdown on ATRA-reversed ALP activities in MEFs. **j** Quantification of ALP staining showed the effect of Serpina3n on ATRA-reversed ALP activities, *p < 0.05, **p < 0.01. GFP, green fluorescent protein; RFP, red fluorescent protein; DMSO, dimethyl sulfoxide; DEX, dexamethasone

Next, we sought to investigate the role of Serpina3n in the reversal caused by ATRA. We constructed adenovirus-mediated overexpression and knockdown models of Serpina3n and validated their functions (Fig. 7a, b). ALP staining and quantification showed that the overexpression of Serpina3n can attenuate ATRA-induced ALP activities (Fig. 7c, d). In contrast, the knockdown of Serpina3n decreased the osteogenic inhibition of DEX with regard to BMP9 in MEFs (Fig. 7e, f). This result was confirmed by Western blot detection of the protein expressions of ALP and RUNX2 (Fig. 7g, h). Then, we combined the overexpression or knockdown of Serpina3n with DEX and ATRA in BMP9-induced osteogenic differentiation. ALP staining and quantification revealed that although the knockdown of Serpina3n hardly enhanced ATRA-reversed ALP activities, overexpression of Serpina3n significantly attenuated the ALP activities reversed by ATRA (Fig. 7i, j). These results indicate that DEX may inhibit BMP9-induced osteogenic differentiation by enhancing the expression of Serpina3n. In addition, ATRA may exert its reversal effect by decreasing Serpina3n.

### Effects of RARβ on the expression of Serpina3n in the reversal process of ATRA with regard to DEX-inhibited osteogenesis

**Fig. 8:**
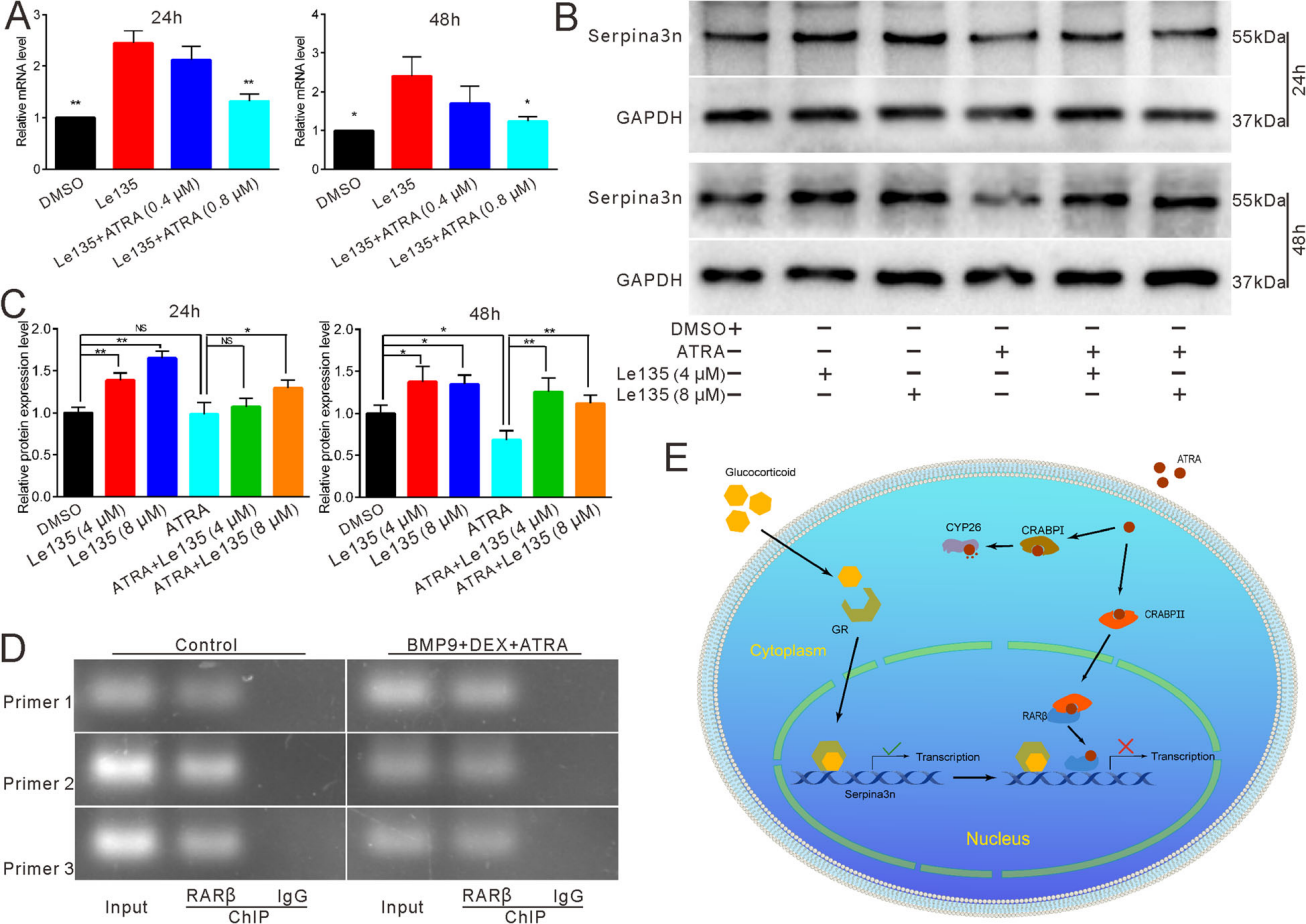
Effects of RARβ on the expression of Serpina3n in the reversal process of ATRA to DEX. **a** RTqPCR results showed the effect of Le135 and ATRA on the expression of Serpina3n, *p < 0.05 vs Le135, **p < 0.01 vs Le135. **b** Western blot results demonstrated the effect of RARβ inhibitor and ATRA on the protein expression of Serpina3n. **c** Quantification of western blot results showed the effect of Le135 and ATRA on the expression of Serpina3n, *p < 0.05, **p < 0.01. **d** Possible reversal mechanism of ATRA on DEX-induced osteogenic differentiation. DEX can be transferred into the nucleus to promote the transcription of Serpina3n; once ATRA is transferred into the nucleus by CRABPII, it will bind to RARβ to further modulate the transcription of Serpina3n. CYP26 is for degradation of ATRA transferred by CRABPI. DMSO, dimethyl sulfoxide; DEX, dexamethasone; Le135, specific inhibitor of RARβ; GR, glucocorticoid receptor; CRABPI, cellular retinoic acid binding protein I; CRABPII, cellular retinoic acid binding protein II; CYP26, cytochrome P450 26

Since RARβ was found to be involved in the reversal process of ATRA, we recruited Le135 to further explore the relationship between ATRA and Serpina3n. RTqPCR results demonstrated that Le135 can promote the expression of Serpina3n and that this effect can be reversed when combined with ATRA (Fig. 8a). Western blot results and quantification showed that Le135 can enhance the expression of Serpina3n in a concentration-dependent manner with or without the combination of ATRA (Fig. 8b, c). Furthermore, ChIP results demonstrated that RARβ can be added to the promoter region of Serpina3n to inhibit its transcription and expression either with or without the mediation of DEX and ATRA (Fig. 8d). The possible graphic mechanism showed that ATRA-activated RARβ blocked the promotion of DEX (glucocorticoid) on Serpina3n to reverse the osteogenic inhibitory effect of DEX (Fig. 8e). These data allude that the reversal effect of ATRA on DEX-inhibited osteogenesis may be achieved by RARβ activation and further Serpina3n inhibition.

## Discussion

In this study, we found that ATRA facilitated BMP9-induced or OIM-induced osteogenic differentiation in different MSC lines, while DEX exerted opposite effects. Therefore, we used BMP9-induced MEFs as an osteogenic model to analyze the relationship between DEX and ATRA. We discerned that ATRA can reverse the inhibitory osteogenic effect of DEX in vitro as well as in vivo in the rat GIOP model. During the reversal process of ATRA, the expression of RARβ was remarkably enhanced. In addition, Le135, the RARβ specific inhibitor, blocked the reversal caused by ATRA, showing that ATRA may exert its function through RARβ. Furthermore, RNA-seq revealed that Serpina3n, the novel gene, was upregulated by DEX but downregulated by ATRA. Meanwhile, knockdown of Serpina3n also reversed DEX-inhibited osteogenic differentiation to some extent, indicating that the reversal effect of ATRA may be achieved by Serpina3n inhibition. ChIP analysis confirmed that RARβ can bind to the promoter region of Serpina3n. Hence, ATRA may reverse DEX-inhibited osteogenic differentiation via RARβ activation and the subsequent inhibition of Serpina3n transcription.

DEX, a synthetic glucocorticoid, has been widely used in the treatment of inflammatory and autoimmune diseases such as systemic lupus erythematosus (SLE), allergies, and rheumatoid arthritis (RA). However, high dosage and long-term use of DEX are associated with severe complications such as GIOP and osteonecrosis of the femoral head (ONFH). Previous studies have asserted that DEX has a dual role in osteogenic differentiation [44]. DEX promotes osteogenesis at the physiological concentration but dramatically inhibits bone metabolism at the pharmacological concentration. Thus, a low concentration of DEX (10^−8^ M) is routinely used in OIM. In our study, the osteogenic potential of DEX was higher at a concentration of 10^−9^ M than at 10^−8^ M (Fig. 2i–l). This observation could be probably because the optimal concentration of DEX that facilitates osteogenic differentiation differs from cell to cell. Our results also proved that a low concentration of DEX enhances osteogenesis while a high concentration exerts the opposite effect (Fig. 2c–h). The effects of DEX on bone metabolism are manifested as osteoblastic inhibition and osteoclastic promotion. DEX induces the apoptosis of osteoblasts and osteocytes by binding to monomeric glucocorticoid receptor (GR); meanwhile, the osteoclasts are activated, leading to an increase in the RANKL/OPG ratio. In addition, adipogenesis is markedly enhanced by DEX in the mesenchymal progenitor cells to compensate the bone loss with adipocytes [2, 44, 45]. Our study showed that DEX decreased the number and thickness of the trabecular bone but increased trabecular separation, which is consistent with previous reports.

Osteogenesis-related studies on ATRA, a derivative of vitamin A, remain controversial. Several studies have reported that ATRA inhibits osteogenic differentiation [46, 47]. However, an increasing number of studies have revealed the effect of ATRA on osteogenic differentiation. Previous studies have established that ATRA can increase the osteogenic ability of BMP9 [32, 43]. Moreover, ATRA also engages in a crosstalk with Wnt/β-catenin to potentiate Wnt3A-induced osteogenic differentiation [43, 48]. Our study showed that ATRA can not only stimulate BMP9-induced osteogenic differentiation (Fig. 1g–k) but also enhance OIM-induced osteogenesis (Fig. 1l, m). However, on its own, ATRA only induced ALP activities but not matrix mineralization in MSCs (Fig. 1a–d and Fig. S1A-B), which agrees with a previous study [49]. Nevertheless, no studies have so far reported the varying effects of ATRA alone on early and late osteogenic markers; the mechanism remains unknown and awaits further investigations. It has been reported that ATRA can bind to RARs and RXRs. However, RXRs are restrictively activated when the concentration of ATRA is higher than 10^−5^ M [29]. As receptors of ATRA, RARs play an important role in bone metabolism. RARγ directly regulates endochondral ossification and indirectly regulates the formation of tibial vessels [50]. RAR agonists attenuate RANKL-mediated osteoclastic differentiation [51]. Besides, RARα acts as an important regulatory component in the differentiation of the mesoderm into chondroblasts [52]. The investigation of RARβ in osteogenic differentiation seems rare. Our study demonstrated that the expression of RARβ was significantly elevated by ATRA in the DEX-inhibited osteogenic model (Fig. 5a) and that Le135 blocked the reversal of ATRA (Fig. 5f–j). Therefore, RARβ may be a crucial receptor in the osteogenic reversal process of ATRA.

Being a novel gene, studies on Serpina3n are limited. Most of the investigations are related to neurology because Serpina3n also acts as a reactive astroglial marker. It has been reported that DEX can remarkably enhance both the gene and protein levels of Serpina3n and that Serpina3n may act as a circulating biomarker for muscle atrophy induced by glucocorticoids [37, 53]. In our study, DEX potentiated the expression of Serpina3n in a concentration-dependent manner (Fig. 6a, b), which is consistent with our RNA-seq results and previous reports involving different cell lines. However, the relationship between Serpina3n and ATRA has not been well explored although ATRA has been shown to be brain protective in a traumatic brain model by partly attenuating the expression of Serpina3n [54]. In our study, ATRA reduced the expression of Serpina3n with or without the mediation of DEX (Fig. 5c, d–g). Furthermore, overexpression of Serpina3n attenuated ATRA-induced ALP activities (Fig. 7c, d), whereas knockdown of Serpina3n partly blocked DEX-inhibited osteogenic differentiation (Fig. 7e, f). Thus, when combined with RARβ, our data strongly suggest that DEX may inhibit BMP9-induced or OIM-induced osteogenic differentiation by enhancing Serpina3n and that ATRA reverses DEX-inhibited osteogenesis by targeting Serpina3n, which is mediated by RARβ.

In our study, we utilized Le135 to explore the role of RARβ. Although the results are convincing, RARβ overexpression or agonists were not introduced or applied in our analysis. This investigation needs to be performed in the future to validate our present results. Furthermore, even though we have investigated the in vitro expressions of Serpina3n during the mediation of DEX and ATRA, the in vivo expressions of Serpina3n and RARβ remain to be tested. In further studies, we plan to focus on Serpina3n using GIOP and ovariectomy models to further explore the role of Serpina3n in GIOP and other osteoporosis. Furthermore, the effects of ATRA on matrix mineralization were inconsistent when compared with the ALP activities induced by ATRA, which requires further investigations.

## Conclusion

Collectively, our findings suggest that ATRA can reverse the inhibitory osteogenic effect of DEX both in vitro and in vivo. ATRA may chiefly activate RARβ and subsequently inhibit the transcription of Serpina3n to antagonize DEX-inhibited osteogenic differentiation. Our results are expected to provide useful insights in the journey of discovering new agents and drugs as well as in identifying the key genes involved in GIOP.

## Supplementary Information

**Additional file 1: Fig. S1.** (A) Alizarin Red S staining showed the effect of different concentrations of ATRA on matrix mineralization in MEFs after 14 days. (B) Quantification of matrix mineralization in MEFs after 14 days, *p < 0.05 vs DMSO, **p < 0.01 vs DMSO. (C) ALP staining showed the effect of different concentrations of ATRA on BMP9-induced ALP activities in C3H10T1/2 cells after 5 and 7 days. (D-E) Quantification of ALP staining showed the effect of different concentrations of ATRA on BMP9-induced ALP activities in C3H10T1/2 cells after 5 and 7 days, *p < 0.05 vs BMP9 + DMSO, **p < 0.01 vs BMP9 + DMSO. (F) ALP staining showed the effect of different concentrations of ATRA on BMP9-induced ALP activities in C2C12 cells after 5 and 7 days. (G-H) Quantification of ALP staining showed the effect of different concentrations of ATRA on BMP9-induced ALP activities in C2C12 cells after 5 and 7 days, *p < 0.05 vs BMP9 + DMSO, **p < 0.01 vs BMP9 + DMSO. (I-J) Alizarin Red S staining and quantification showed the effect of different concentrations of ATRA on BMP9-induced matrix mineralization in MEFs after 21 days, *p < 0.05 vs BMP9 + DMSO, **p < 0.01 vs BMP9 + DMSO.

**Additional file 2: Fig. S2.** (A) ALP staining showed the effect of different concentrations of DEX on BMP9-induced ALP activities in C3H10T1/2 cells after 5 and 7 days. (B-C) Quantification of ALP staining showed the effect of different concentrations of DEX on BMP9-induced ALP activities in C3H10T1/2 cells after 5 and 7 days, *p < 0.05 vs BMP9 + DMSO, **p < 0.01 vs BMP9 + DMSO. (D) ALP staining showed the effect of different concentrations of DEX on BMP9-induced ALP activities in C2C12 cells after 5 and 7 days. (E-F) Quantification of ALP staining showed the effect of different concentrations of DEX on BMP9-induced ALP activities in C2C12 cells after 5 and 7 days, *p < 0.05 vs BMP9 + DMSO, **p < 0.01 vs BMP9 + DMSO.

**Additional file 3: Fig. S3.** (A) ALP staining showed the effect of different concentrations of ATRA on different concentrations of DEX-inhibited ALP activities in C3H10T1/2 cells after 5 days. (B-E) Quantification of ALP staining showed the effect of different concentrations of ATRA on different concentrations of DEX-inhibited ALP activities, *p < 0.05, **p < 0.01.

## Data Availability

All datasets used and analyzed in study are available from the corresponding author on reasonable request.

## Abbreviations

ATRA: All-trans retinoic acid
DEX: Dexamethasone
Serpina3n: Serine protease inhibitor 3n
MSCs: Mesenchymal stem cells
RARβ: Retinoic acid receptor β
GFP: Green fluorescent protein
RFP: Red fluorescent protein
BMP9: Bone morphogenetic protein 9
ALP: Alkaline phosphatase
RUNX2: Runt-related transcription factor 2
OPN: Osteopontin
OCN: Osteocalcin
DMSO: Dimethyl sulfoxide
DMEM: Dulbecco’s modified Eagle’s medium
OIM: Osteogenic inductive medium
GR: Glucocorticoid receptor
CRABPI: Cellular retinoic acid binding protein I
CRABPII: Cellular retinoic acid binding protein II
CYP26: Cytochrome P450 26
OVX: Ovariectomy
