# Supplementary figures and images for: The role of Serpina3n in the reversal effect of ATRA on dexamethasone-inhibited osteogenic differentiation in mesenchymal stem cells
Source: Stem Cell Res Ther. 2021 May 17;12:291. doi: 10.1186/s13287-021-02347-0 (PMC8127316; doi:10.1186/s13287-021-02347-0)

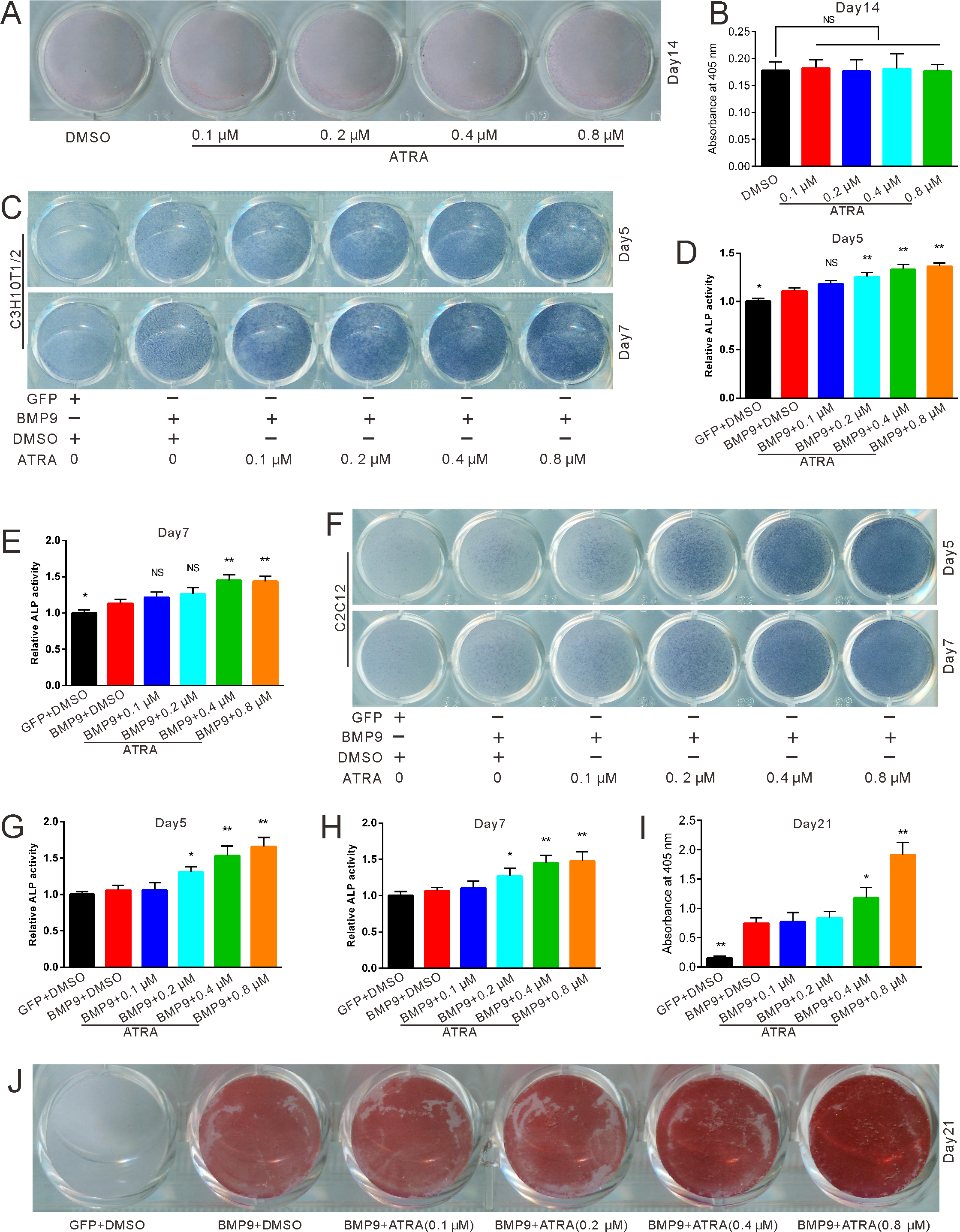

Supplement: Supplementary file 1 — Additional file 1: Fig. S1. (A) Alizarin Red S staining showed the effect of different concentrations of ATRA on matrix mineralization in MEFs after 14 days. (B) Quantification of matrix mineralization in MEFs after 14 days, *p < 0.05 vs DMSO, **p < 0.01 vs DMSO. (C) ALP staining showed the effect of different concentrations of ATRA on BMP9-induced ALP activities in C3H10T1/2 cells after 5 and 7 days. (D-E) Quantification of ALP staining showed the effect of different concentrations of ATRA on BMP9-induced ALP activities in C3H10T1/2 cells after 5 and 7 days, *p < 0.05 vs BMP9 + DMSO, **p < 0.01 vs BMP9 + DMSO. (F) ALP staining showed the effect of different concentrations of ATRA on BMP9-induced ALP activities in C2C12 cells after 5 and 7 days. (G-H) Quantification of ALP staining showed the effect of different concentrations of ATRA on BMP9-induced ALP activities in C2C12 cells after 5 and 7 days, *p < 0.05 vs BMP9 + DMSO, **p < 0.01 vs BMP9 + DMSO. (I-J) Alizarin Red S staining and quantification showed the effect of different concentrations of ATRA on BMP9-induced matrix mineralization in MEFs after 21 days, *p < 0.05 vs BMP9 + DMSO, **p < 0.01 vs BMP9 + DMSO. [file 13287_2021_2347_MOESM1_ESM.tif]

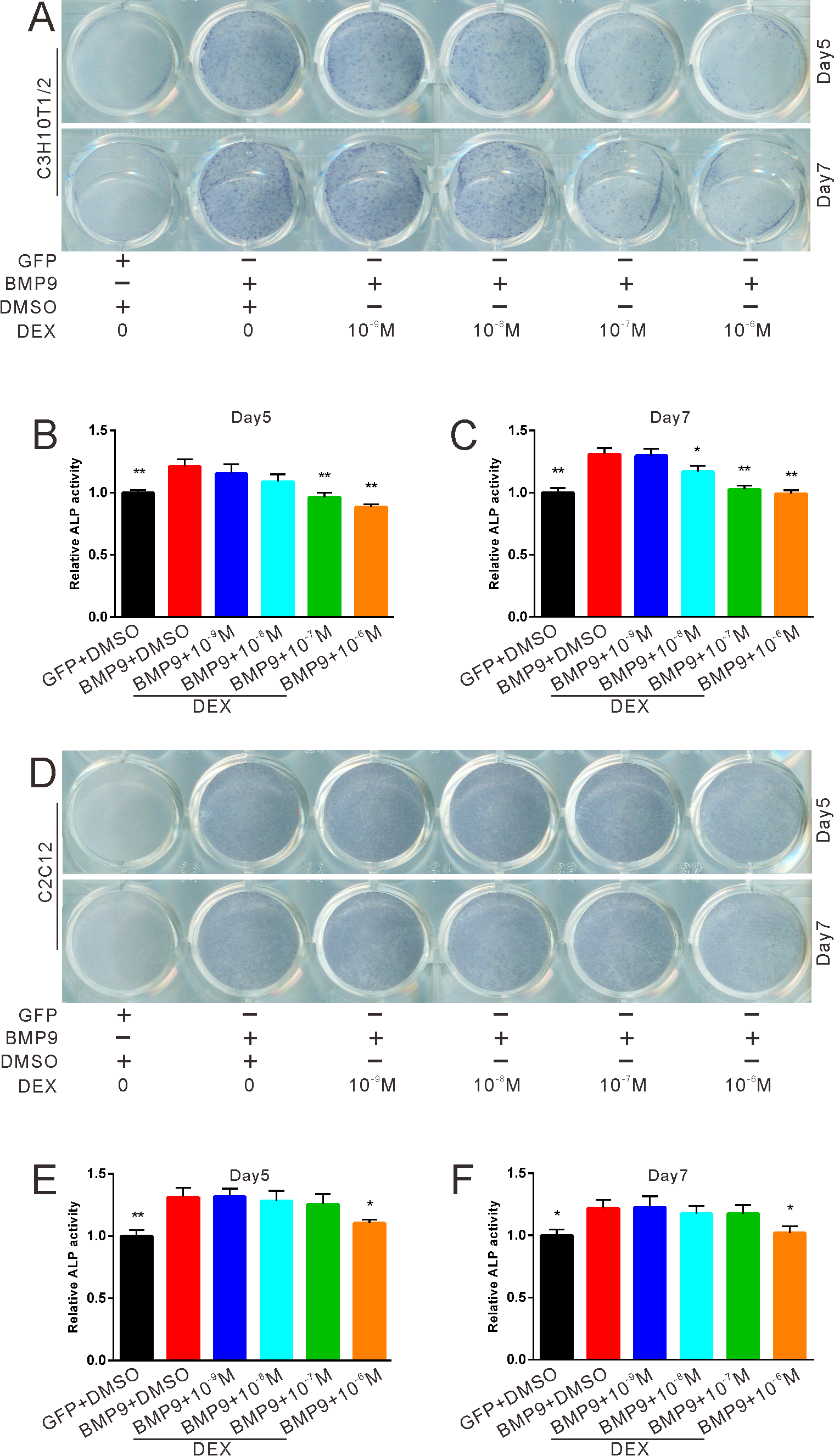

Supplement: Supplementary file 2 — Additional file 2: Fig. S2. (A) ALP staining showed the effect of different concentrations of DEX on BMP9-induced ALP activities in C3H10T1/2 cells after 5 and 7 days. (B-C) Quantification of ALP staining showed the effect of different concentrations of DEX on BMP9-induced ALP activities in C3H10T1/2 cells after 5 and 7 days, *p < 0.05 vs BMP9 + DMSO, **p < 0.01 vs BMP9 + DMSO. (D) ALP staining showed the effect of different concentrations of DEX on BMP9-induced ALP activities in C2C12 cells after 5 and 7 days. (E-F) Quantification of ALP staining showed the effect of different concentrations of DEX on BMP9-induced ALP activities in C2C12 cells after 5 and 7 days, *p < 0.05 vs BMP9 + DMSO, **p < 0.01 vs BMP9 + DMSO. [file 13287_2021_2347_MOESM2_ESM.tif]

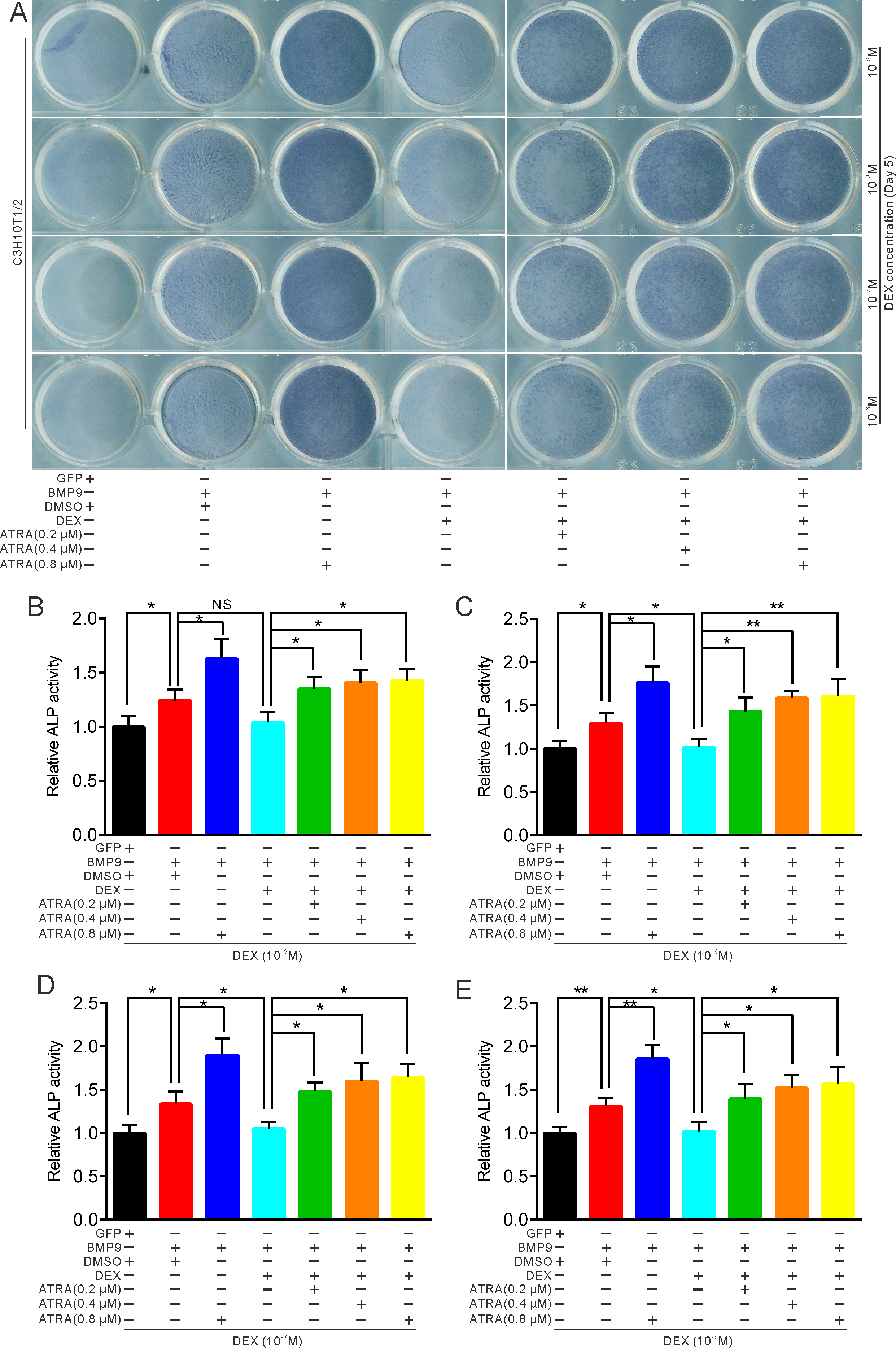

Supplement: Supplementary file 3 — Additional file 3: Fig. S3. (A) ALP staining showed the effect of different concentrations of ATRA on different concentrations of DEX-inhibited ALP activities in C3H10T1/2 cells after 5 days. (B-E) Quantification of ALP staining showed the effect of different concentrations of ATRA on different concentrations of DEX-inhibited ALP activities, *p < 0.05, **p < 0.01. [file 13287_2021_2347_MOESM3_ESM.tif]
